# Substrate recognition of holocytochrome *c* synthase: N-terminal region and CXXCH motif of mitochondrial cytochrome *c*

**DOI:** 10.1016/j.febslet.2014.07.026

**Published:** 2014-09-17

**Authors:** Yulin Zhang, Julie M. Stevens, Stuart J. Ferguson

**Affiliations:** Department of Biochemistry, University of Oxford, South Parks Road, Oxford OX1 3QU, United Kingdom

**Keywords:** HCCS, holocytochrome *c* synthase, Ccm, cytochrome *c* maturation, Cytochrome *c*, Holocytochrome *c* synthase, Heme, Ccm system

## Abstract

Holocytochrome *c* synthase (HCCS) attaches heme covalently to mitochondrial respiratory cytochromes *c*. Little is known about the reaction of heme attachment to apocytochromes *c* by HCCS, although recently it has been established that the CXXCH motif and the N-terminus of the apocytochrome polypeptide are important protein–protein recognition motifs. Here, we explore further the important features of the N-terminal sequence and investigate what variations in the CXXCH residues are productively recognised by HCCS in its substrate.

## Highlights

• Holocytochrome c synthase (HCCS) does not attach heme to cytochromes lacking the histidine in the CXXCH motif.
• HCCS can recognise C-terminally truncated cytochromes *c*.
• The aromatic nature of, or possibly shape complementarity to, F15 in cytochrome *c* is important for recognition by HCCS.
• The spacing of the phenylalanine relative to the CXXCH is a recognition feature.

## 1 Introduction

The biosynthesis of cytochrome *c* is achieved by different systems in different organisms [1]. The heme group is covalently attached to apocytochrome in a critical and poorly understood step in this post-translational modification process. A characteristic and highly conserved CXXCH motif occurs in the cytochrome amino acid sequence; the two cysteine side chains are covalently linked to the vinyl groups of the heme to form holocytochrome *c*. The histidine in the motif usually acts as a ligand to the iron in the heme. In the mitochondria of yeast and animals a single protein attaches heme to cytochrome *c*; the protein is called holocytochrome *c* synthase (HCCS) and is also referred to as cytochrome *c* biogenesis System III or heme lyase. HCCS functions in the mitochondrial intermembrane space, upon import of the apocytochrome from the cytosol [2]. The import of apocytochrome *c*, coordinated by HCCS, has a close relationship with the heme attachment. There is an absence of signal sequence cleavage from the protein in the process of import, except the N-terminal methionine [3]. Although apocytochrome *c* is able to reversibly enter and exit through the mitochondrial outer membrane [4], several studies show that there is an interaction between the apocytochrome *c* and HCCS to form a complex [5], [6].

The mechanism whereby the mitochondrial HCCS attaches heme to apocytochrome remained largely uninvestigated for many years, save for a finding that it would not attach heme to structurally related bacterial *c*-type cytochromes and that one or more CP motifs (a cysteine-proline sequence has often been considered as a heme binding motif in several proteins) in the HCCS sequence were found to be important for HCCS function [7]. The situation has changed in the past three years because the importance of the CP motifs has been questioned [8] and several studies have shown that the N-terminal region of the mitochondrial cytochrome *c* sequence is critical for attachment of heme [9], [10], [11], [12]. Arguably surprisingly, a conserved phenylalanine at position 15 in the yeast protein and 11 in horse heart, but in each case separated by only three residues from the CXXCH motif*,* and for many years thought only to be essential for cytochrome *c* structural integrity, has been shown to be essential for heme attachment but not necessary for properly folded protein. This has left open questions including whether phenylalanine in this position is absolutely essential in the sense of both the nature of the residue and its exact position relative to the CXXCH motif. It was especially notable that the cytochrome *c* of *Paracoccus denitrificans*, which is not processed by HCCS, has a phenylalanine residue spaced only one residue differently than in mitochondrial cytochromes *c* [13].

Some previous studies have focused on the importance of the complete CXXCH motif which is near to the N-terminus in mitochondrial cytochromes *c*. The two cysteine residues and the histidine residue in the motif are generally highly conserved with a few exceptions. There have been reports of both success [14], [15] and failure [16] of heme attachment to SXXCH and CXXSH variants of yeast and human cytochrome *c*. A CXXCR variant of yeast cytochrome *c* was also reported to be able to be expressed by plasmid-based expression systems, but not by the native cytochrome biosynthetic system of yeast itself [17], [18], [19].

In the prokaryotic world, various heme-containing proteins have motifs different from CXXCH motif, such as the CXXCK motif of NrfA, a bacterial nitrite reductase. Analysis of the CXXCK, CXXCM and CXXCR variants of a double-cysteine variant of cytochrome *b*_562_ had been carried out with the cytochrome *c* maturation (Ccm) system that is found in many Gram-negative bacteria including *Escherichia coli*; all the variants failed to mature [20]. There are also studies reporting the outcome of varying the number of residues between the two cysteines or mutating these residues [11], [21], with mixed results. There is less information about the tolerance of HCCS to similar changes in its substrate.

Here we utilise well-established systems with either one-plasmid or two-plasmid methods of HCCS-cytochrome *c* co-expression in *E. coli* [22], [23] for the investigation of tolerance of absence of the cysteine and histidine residues in the CXXCH motif and of variations in the N-terminal sequence. In this type of approach a failure to observe heme attachment to a variant cytochrome could result from either failure of catalysis by HCCS, severe instability of the apo proteins or attachment of heme followed by rapid degradation of unstable protein. We and others [11], [12] introduced the control of seeking to mature the same cytochrome variants in the *E. coli* periplasm using the Ccm system, or System I, which has much broader substrate specificity than System III; it is able to carry out heme attachment under most circumstances when a CXXCH motif is present [24] irrespective of neighbouring residues. By applying this strategy to cytochrome *c* variants, we thus have a way to distinguish HCCS substrate recognition properties from the features responsible for structural integrity of the cytochrome *c* protein. In addition, there are examples of heme attachment by mitochondrial extracts [25] to cytochrome *c* fragments [9]. In this context, we also investigated several truncations of *Saccharomyces cerevisiae* cytochrome *c* to test for heme attachment to these fragments by HCCS. The present paper describes experiments that address these issues, some of which have also been addressed in complementary ways in other recent papers to which we return in the Discussion. There is also some ambiguity as to which residues in the CXXCH motif are essential for processing, with reports that variation at the H is possible and that either of the cysteine residues can be changed. These issues are also considered in the present paper.

## 2 Material and methods

### 2.1 Plasmid and strains

A system with HCCS and cytochrome *c* on two different plasmids (two-plasmid system), from which iso-1 cytochrome *c* (CYC1) and HCCS from *S. cerevisiae* are expressed from separate plasmids, as well as a system with both genes on one plasmid (single-plasmid system) in which the horse cytochrome *c* and *S. cerevisiae* HCCS are encoded on the same plasmid, were used [13], [23]. The single-plasmid system has been optimised for high levels of production of horse holocytochrome *c,* facilitating detection and analysis of the product. The two-plasmid system has the advantage that both genes are from the same organism which might simplify interpretation of interactions between the two proteins. Nonetheless, yeast HCCS matures both yeast and horse cytochromes *c*. The QuikChange® Site-Directed Mutagenesis Kit with KOD polymerase (Stratagene) was used with the wild-type plasmids (see Table 1) as templates to generate the variants. The constructs were all sequenced (Geneservice, Oxford) and then transformed into *E. coli* strain BL21(DE3). A list of the constructs and strains is shown in Table 1.

**Table 1:** List of strains and plasmids used in this work.

Name	Description	Source
pOScyc1	*S. cerevisiae* cyc1, Amp^R^	[13]
pWT cytochrome *c*	*S. cerevisiae* cyc3, *Equus caballus* CYCS, Amp^R^	[23]
pYZ05	pOScyc1 with F15A mutation, Amp^R^	[12]
pYZ07	pWT with C15A mutation	This work
pYZ08	pWT with C18A mutation	This work
pYZ09	pWT with H19K mutation	This work
pYZ10	pWT with H19M mutation	This work
pYZ11	pWT with H19R mutation	This work
pYZ12	pOScyc1 with F15Y mutation, Amp^R^	This work
pYZ13	pOScyc1 with F15W mutation, Amp^R^	This work
pYZ14	pOScyc1 with F15I mutation, Amp^R^	This work
pYZ15	pOScyc1 with F15E mutation, Amp^R^	This work
pYZ16	pOScyc1 with T17 deletion, Amp^R^	This work
pYZ17	pOScyc1 with K16 deletion, Amp^R^	This work
pYZ18	pOScyc1 with G29 truncation, Amp^R^	This work
pYZ19	pOScyc1 with H45 truncation, Amp^R^	This work
pYZ20	pOScyc1 with K60 truncation, Amp^R^	This work
pOScyc1peri	pOScyc1 with periplasmic targeting sequence, Amp^R^	This work
pYZ21	pOScyc1peri with F15A mutation, Amp^R^	This work
pYZ22	pOScyc1peri with F15Y mutation, Amp^R^	This work
pYZ23	pOScyc1peri with F15W mutation, Amp^R^	This work
pYZ24	pOScyc1peri with F15I mutation, Amp^R^	This work
pYZ25	pOScyc1peri with F15E mutation, Amp^R^	This work
pYZ26	pOScyc1peri with T17 deletion, Amp^R^	This work
pYZ27	pOScyc1peri with K16 deletion, Amp^R^	This work

### 2.2 Bacterial cultures and cell fractionation

Horse heart cytochrome *c* or Strep-tagged yeast cytochrome *c* were expressed in *E. coli* strain BL21(DE3) with yeast HCCS. Periplasmically targeted Strep-tagged yeast cytochrome was constructed by the addition of the *Pseudomonas aeruginosa* cytochrome *c*_551/552_ pre-sequence to pOScyc1. The resulting plasmid was then co-expressed with the *E. coli* cytochrome *c* maturation system genes on plasmid pEC86 in BL21(DE3) [26]. Isopropyl β-d-1-thiogalactopyranoside (IPTG) was used as the induction agent. Cells were cultured in LB (Luria–Bertani) media or 2-YT media supplemented with 100 μg/ml ampicillin (and 33 μg/ml chloramphenicol in the case of the two-plasmid system) for 20 h in a 30 °C incubator with 200 rpm shaking. 125 ml cultures in 250 ml flask were used in most experiments. 500 ml cultures in 2 l flasks were used when a larger quantity of cell extract was required for analysis. The periplasm was separated to produce spheroplasts using a procedure described previously [27]. Cytoplasmic extraction was performed by sonication of the spheroplasts and 35 min centrifugation at 16 000×*g*.

### 2.3 Cytochrome analysis

Cell extracts were analysed by SDS–PAGE gel electrophoresis (pre-cast 10% Bis-Tris gels, NuPage) followed by heme-staining as described [28]. UV–vis spectroscopy was performed on extracts using a Perkin Elmer UV–visible Lambda 2 spectrometer. A 19% (vol/vol) pyridine solution in 0.15 M NaOH was used to determine pyridine hemochrome spectra [29]. The position of the α-band in the visible spectra of denatured hemoproteins in a pyridine solution is indicative of the saturation of the heme vinyl groups and therefore whether no, one or two heme-protein thioether bonds are present.

### 2.4 Western blots

Western blots were used to determine the level of expression of cytochrome *c* in cell extracts. A conjugated Strep alkaline phosphatase antibody (IBA) was used for detection. SIGMAFAST BCIP/NBT tablets were used for development.

## 3 Results

### 3.1 Substitutions within the CXXCH motif of mitochondrial cytochrome *c*

**Fig. 1:**
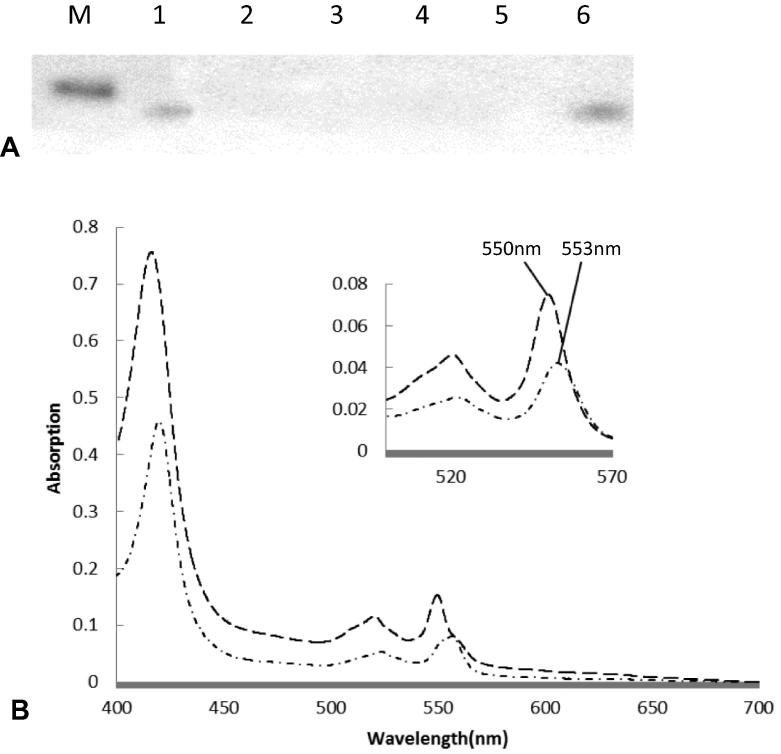
(A) Heme Stain SDS–PAGE analysis of cytoplasmic extracts of wild-type and cysteine or histidine variants within the CXXCH motif of *E. caballus* cytochrome *c*. Equal volumes of cytoplasmic extracts – diluted so as to contain equal amounts of wet cell mass – were loaded on each lane. The 14 kDa molecular marker is shown in lane M. Lane 1 shows wild-type yeast cytochrome *c*, lane 2 the H19M variant, lane 3 the H19K variant, lane 4 the H19R variant, lane 5 the C18A variant, and lane 6 shows the C15A variant. (B) UV–vis absorption spectra of similar volumes of cytoplasmic extracts of wild-type (long dashed line) and C15A variant (dashed-dot line) of *E. caballus* cytochrome *c*. The reduced spectra are shown as the main figure and the inset shows the α-band region of pyridine hemochrome on an expanded scale.

The cysteine residues and the histidine residue in the CXXCH of the cytochrome *c* of *Equus caballus* were substituted with alanine, lysine, methionine or arginine producing the variants C15A, C18A, H19K, H19M and H19R. These were co-expressed with *S. cerevisiae* HCCS and analysed by heme-stained SDS–PAGE gels, as shown in Fig. 1 (A). The C15A variant (AXXCH) alone was matured, as seen by a heme-staining band at the expected molecular mass, and all the other variants did not undergo heme attachment. The gels were overloaded to maximise detection. The level of holocytochrome *c* for the C15A variant is lower than wild-type according to the intensity of the absorption spectra normalised for cell mass. According to UV–vis spectra, as shown in Fig. 1 (B), the wavelengths of the absorption maxima in the pyridine hemochrome spectra were shifted, *i.e*. the α peak shifted from 550 nm to 553 nm, as would be expected for a cytochrome *c* with a single bond between the heme and the protein.

### 3.2 Truncation of the C-terminal sequence of mitochondrial cytochrome *c*

**Fig. 2:**
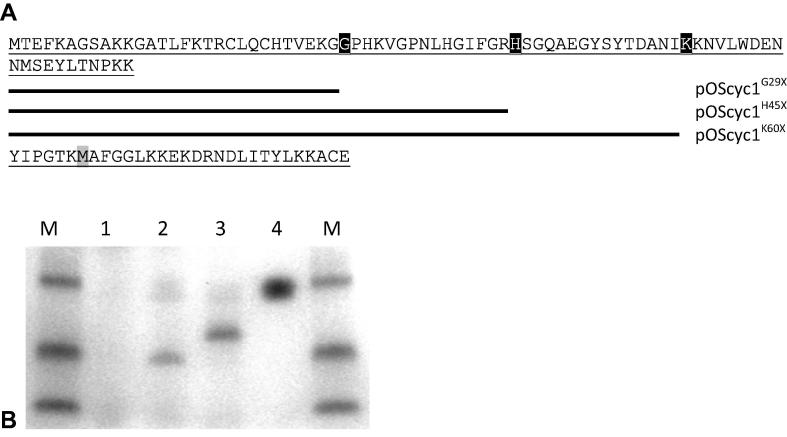
(A) Amino acid sequence of *S. cerevisiae* cytochrome *c* iso-1 and representations of the truncated N-terminal fragments of it with C-termini at residues G29, H45 and K60 (all coloured in black boxes). The heme axial ligand residue M86 is depicted in a grey box. (B) Heme-stained SDS–PAGE analysis of cytoplasmic extracts of wild-type and C-terminal truncation variants of *S. cerevisiae* cytochrome *c*. Equal volumes of cytoplasmic extract (diluted so as to contain equal amounts of wet cell mass) were loaded on each lane. Lane M shows protein markers (3, 6 and 14 kDa from the bottom). Lane 1 shows the G28X variant of yeast cytochrome *c*, lane 2 the H45X variant, lane 3 the K60X variant and lane 4 shows the wild-type cytochrome *c*. Note that the wild-type cytochrome *c* was 100-fold diluted.

A stop codon was introduced in different positions in the gene of *S. cerevisiae* iso-1 cytochrome *c* (CYC1) to remove different lengths of the C-terminal region, producing three variants: G29X, H45X and K60X. The X indicates the position of the inserted stop codon, as indicated on the sequence alignments in Fig. 2 (A). The truncated variants and the wild type protein were co-expressed with *S. cerevisiae* HCCS in *E. coli*; Fig. 2 (B) shows the results of heme-stained SDS–PAGE analysis of cytoplasmic extracts. Heme-staining bands were observed with predicted molecular masses (∼6.5 kDa and ∼8 kDa) for H45X and K60X variants, but no reproducible product for the shortest variant, G29X, was detected. The low levels of heme-containing truncations can be ascribed to relative instability of these molecules.

### 3.3 Substitution of a single phenylalanine in the N-terminal region of mitochondrial cytochrome *c*

**Fig. 3:**
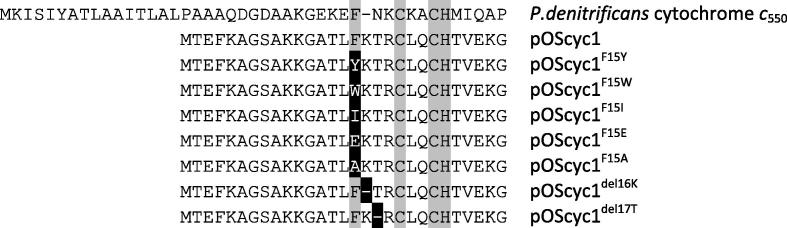
N-terminal sequences of *S. cerevisiae* cytochrome *c* iso-1 and its variants with *P. denitrificans* cytochrome *c*_550_ for comparison. Highly conserved amino acid residues are depicted in grey boxes, and the mutations are depicted in black boxes.

**Fig. 4:**
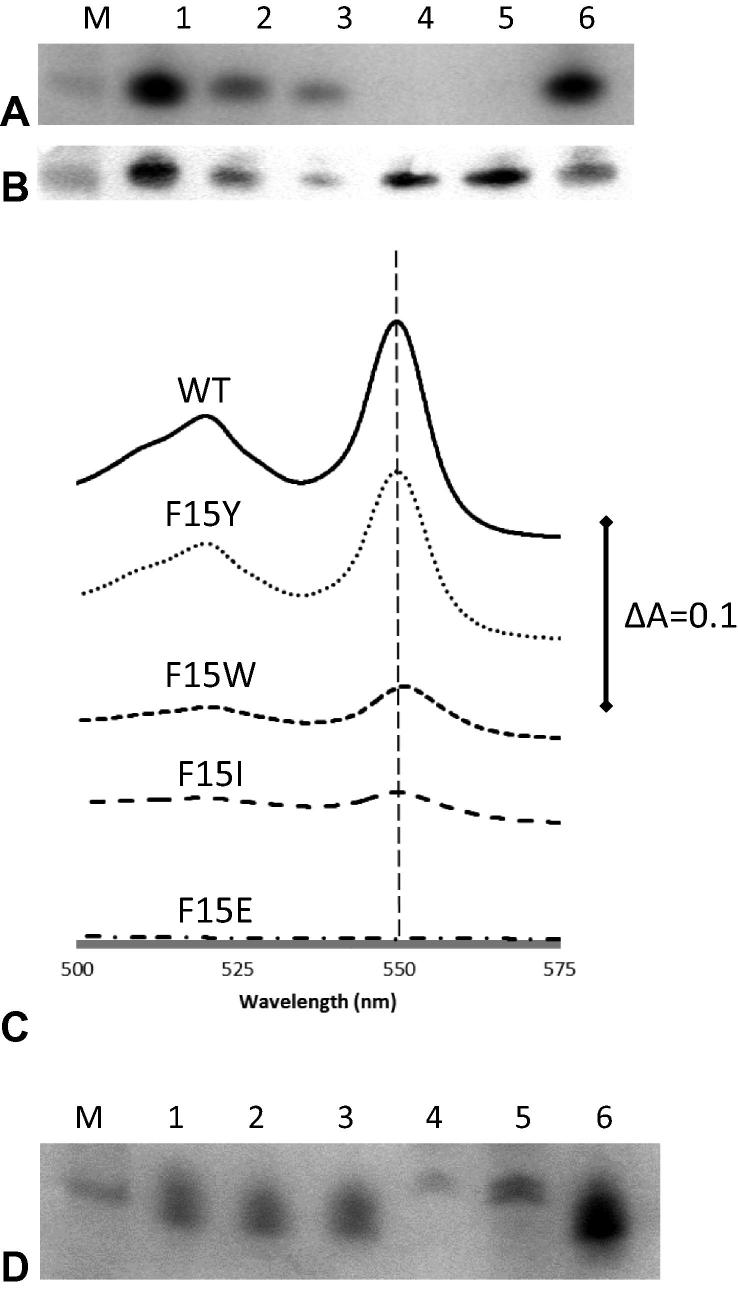
(A) Heme-stained SDS–PAGE analysis of cytoplasmic extracts of wild-type and phenylalanine 15 variants of *S. cerevisiae* cytochrome *c*. The volumes of cytoplasmic extract loaded on each lane are equal and normalised according to wet cell mass. Lane M shows the 14 kDa molecular marker. Lane 1 shows the F15Y variant of yeast cytochrome *c*, lane 2 the F15W variant, lane 3 the F15I variant, lane 4 the F15E variant, lane 5 the F15A variant and lane 6 shows the wild-type cytochrome *c*. (B) Western Blot of cytoplasmic extracts of wild-type and phenylalanine 15 variants of *S. cerevisiae* cytochrome *c*. An anti-Strep antibody was used in the blotting. The volumes of cytoplasmic extract loaded in each lane are equal (diluted so as to contain equal amounts of wet cell mass). Lane M shows the 14 kDa molecular marker. Lane 1 shows the F15Y variant of yeast cytochrome *c*, lane 2 the F15W variant, lane 3 the F15I variant, lane 4 the F15E variant, lane 5 the F15A variant and lane 6 shows the wild-type cytochrome *c*. (C) α-band region of pyridine hemochrome UV–vis absorption spectra of reduced cytoplasmic extracts of wild-type and phenylalanine 15 variants of *S. cerevisiae* cytochrome. (D) Heme stain SDS–PAGE analysis of periplasmic extracts of periplasmically targetted wild-type and phenylalanine 15 variants of *S. cerevisiae* cytochrome *c*. Lane M shows the 14 kDa molecular marker. Lane 1 shows the F15Y variant of yeast cytochrome *c*, lane 2 the F15W variant, lane 3 the F15I variant, lane 4 the F15E variant, lane 5 the F15A variant and lane 6 shows the wild-type cytochrome *c*.

We explored further what, if any, other residues, could substitute for the crucial F15, identified by us and others as key for recognition by HCCS. Four additional replacements (Fig. 3) of the phenylalanine in the N-terminal region of *S. cerevisiae* cytochrome *c* were made (F15Y, F15W, F15I and F15E). A F15A variant (which was reported in previous work [12]) was also included in this analysis for comparison with the new variants. The results of SDS–PAGE analysis and UV–vis absorption spectra in Fig. 4 (A) and (C) show that the F15A and F15E replacements abolished holocytochrome *c* production completely, whereas the maturation of cytochrome *c* is attenuated, but not abolished, to different extents in both the F15W and F15I variants. The F15Y variant retains a high level of heme attachment. This implies the aromatic R-group of residue F15 is important in recognition by HCCS, as replacement of phenylalanine by both tryptophan and tyrosine had smaller impacts on the level of heme attachment than replacement with other residues including the hydrophobic isoleucine. This might indicate the importance of involvement of an aromatic residue in a π–π or π-cation interaction. The wavelengths of the peaks in the α-band region of the pyridine hemochrome UV–vis absorption spectra of the variants were at 550 nm as for the wild type, as shown in Fig. 4 (C), indicating that the two heme-to-protein thioether bonds had formed in these cases. However, the Western blot result in Fig. 4 (B) shows that while F15A and F15E variants produce no holocytochrome *c*, there was a significant level of apocytochrome *c* produced in the cytoplasmic extracts for both variants. On the contrary, the overall cytochrome *c* (apo and holo) levels of F15W and F15I variants are lower than the others. One possibility for the abolition of the holocytochrome *c* production for F15E and F15A could be due to intrinsic structural disturbance introduced by the replacements, instead of the failure of recognition by HCCS. The observation of readily detectable levels of apocytochrome of the F15A and F15E variants in cytoplasmic extracts suggests that the lack of heme attachment to these two variants is caused by loss of recognition by HCCS, rather than structural instability of the proteins in either the apo or holo forms.

In order to further demonstrate the importance of the F15 residue in the context of recognition but not structural significance, a periplasmically targeted yeast cytochrome *c* was constructed and tested by co-expressing with the Ccm system from the plasmid pEC86. The 23-amino-acid long periplasmic targeting sequence MKPYALLSLLATGTLLAQGAWAED from *P. aeruginosa* cytochrome *c*_551/552_ was inserted at the beginning of the sequence of Strep-tagged *S. cerevisiae* cytochrome *c*. Periplasmically targeted variants F15A, F15Y, F15W, F15I and F15E were made from this construct. Despite efforts to optimise the procedure, periplasmic extraction of the wild-type and variants were incomplete as large amounts of periplasmically targeted cytochrome remained as contaminants in the cytoplasmic portion after the extraction. All periplasmically targeted variants, except F15E, expressed readily detectable quantities of holocytochrome *c*. Fig. 4 (D) shows SDS–PAGE heme-staining analysis of whole-cell extracts for the five variants and wild-type cytochrome *c*. The periplasmically targeted F15I variant was matured to a similar level as the F15Y and F15W variants. The larger size of F15Aperi and F15Eperi is attributed to incomplete cleavage of the targeting signal sequence as observed previously [12]. Both of them also show a much lower level of production of holocytochrome *c* than wild-type. The key point here is that the Ccm apparatus of *E. coli* that functions in the periplasm essentially recognises only the CXXCH motif and thus variant mitochondrial holocytochrome *c* with F15A and F15E can be formed, whereas the HCCS system is unable to process these variants. Taken together with the detection of apo F15A and F15E proteins, this is strong evidence that the requirement for a particular residue at position 15 is a consequence of the demands of HCCS and not the demands for structural integrity of holocytochrome *c*.

### 3.4 Deletion of amino acids adjacent to the phenylalanine the N-terminal region of mitochondrial cytochrome *c*

**Fig. 5:**
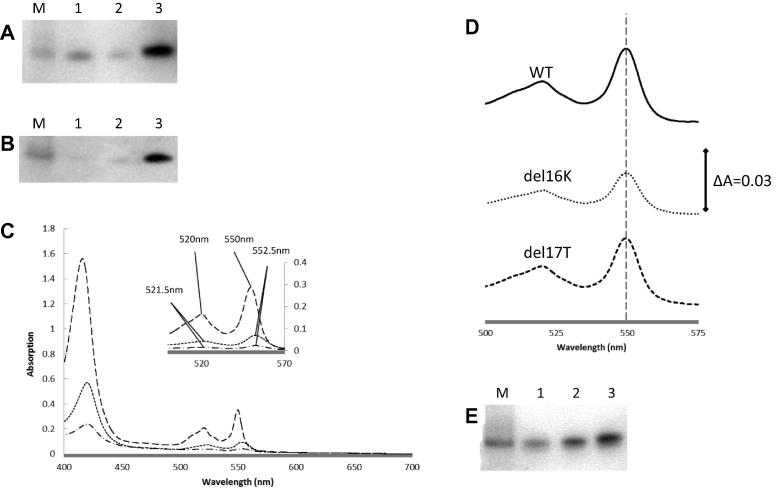
(A) Heme-stained SDS–PAGE analysis of cytoplasmic extracts of wild-type and N-terminal deletion variants of *S. cerevisiae* cytochrome *c*. Equal volumes of cytoplasmic extract were loaded on each lane and normalised according to wet cell mass. Lane M shows the protein marker 14 kDa. Lane 1 show the del16K variant of yeast cytochrome *c*, lane 2 the del17T variant and lane 3 show the wild-type cytochrome *c*. (B) Western Blot of cytoplasmic extracts of wild-type and deletion variants of *S. cerevisiae* cytochrome *c*. The volumes of cytoplasmic extract loaded on each lane are equal (diluted so as to contain equal amounts of wet cell mass). Lane M shows molecular markers 14 kDa. Lane 1 show the del16K variant of yeast cytochrome *c*, lane 2 the del17T variant and lane 3 show the wild-type cytochrome *c*. (C) UV–vis absorption spectra of cytoplasmic extracts of wild-type (long dashed line), delK16 (dotted line) and delT17 (dashed-dot line) of *S. cerevisiae* cytochrome *c*. The reduced spectra are shown as the main figure and the inset shows the α-band region of the pyridine hemochrome spectra on an expanded scale. (D) α-Band region of pyridine hemochrome UV–vis absorption spectra of reduced periplasmic extracts of periplasmically targeted wild-type and deletion variants of *S. cerevisiae* cytochrome. (E) Heme-stained SDS–PAGE analysis of periplasmic extracts of wild-type and N-terminal deletion variants of periplasmically targeted *S. cerevisiae* cytochrome *c*. Lane M shows the protein marker 14 kDa. Lane 1 shows the del16K variant of yeast cytochrome *c*, lane 2 the del17T variant and lane 3 shows the wild-type cytochrome *c*.

Amino acid R18 in *S. cerevisiae* cytochrome *c* is a highly conserved residue, and is directly adjacent to the CXXCH motif in the amino acid sequence on the N-terminal side. Its significance in HCCS substrate recognition was analysed in previous work. The equivalent residue to this arginine in the N-terminal region in *P. denitrificans c*_550_ is a lysine which has an adjacent asparagine residue; however, in *S. cerevisiae* cytochrome *c* there are three residues (KTR) between the phenylalanine F15 and CXXCH motif but in *P. denitrificans* just two residues (NK). The sequence alignment of two deletion variants with the wild type *S. cerevisiae* cytochrome *c* and the *P. denitrificans c*_550_ is shown in Fig. 3. To test the importance of this spacing difference and its possible implication in the substrate recognition by HCCS, two variants del16K and del17T in yeast cytochrome *c* were made by deleting either lysine or threonine. Fig. 5 shows the heme-stained SDS–PAGE, UV–vis absorption spectra and Western Blot analysis of the cytoplasmic extracts of cells expressing the deletion variants co-expressed with yeast HCCS. Neither of them had heme attachment completely abolished; however, the levels were significantly diminished. The total cytochrome *c* levels of the two deletion variants are also significantly lower than wild-type, according to the Western Blot in Fig. 5 (B).

The pyridine hemochrome UV–vis spectra in Fig. 5 (C, inset) shows while the levels of the two deletion variants are significantly lower than that of wild-type, the wavelengths of the absorption maxima of the variants are shifted from 550 nm (wild-type) to 552.5 nm (α peak).

As it was necessary to examine the possible role of intrinsic instability in the variants, the same deletion variants were also made in the periplasmically targeted *S. cerevisiae* cytochrome *c.* These were co-expressed with the Ccm system from pEC86. In Fig. 5 (D), the wavelengths of the peaks in the α-band region of the pyridine hemochrome UV–vis absorption spectra of the variants are same as the wild type value at 550 nm. The heme-stained SDS–PAGE analysis of cytoplasmic extracts results shows that both of the variants are produced in considerable amounts by the Ccm system as shown in Fig. 5 (E). The Ccm apparatus is able to mature the deletion variants of yeast cytochrome *c* without a change in the nature of the heme binding. Thus cytochrome *c* seems capable of folding to a stable structure once heme is added to the deletion proteins by the Ccm system. Attempts at the reciprocal experiment, addition of an alanine residue to the *P. denitrificans* cytochrome *c*_550_ to increase the spacing between F and CXXCH to three, did not result in the production of holocytochrome *c* in detectable amount (not shown).

In summary, HCCS appears to have misattached heme to the two deletion mutants but when the same two proteins were expressed in the periplasm, the Ccm system processed them ‘normally’ as judged by both a pyridine hemochrome spectra and relative intensities on a heme-stained gel. Thus the cytochrome *c* itself does not appear to be compromised by the deletions but rather it is the action of HCCS that is affected. The misattachment is most likely the formation of a single thioether bond rather than the usual two in the HCCS-processed proteins.

## 4 Discussion

### 4.1 Requirements for maturation in the CXXCH motif of *E. caballus* cytochrome *c*

This work confirms conclusions from several previous studies which showed that HCCS can attach heme to an AXXCH motif and supports the majority of studies [14], [15], [30] but very little or no heme attachment to CXXAH motif. The visible absorption spectra of the AXXCH variant are consistent with a single thioether bond and this variant is comparable with the wild type *c*-type cytochromes from trypanosomes. The absence of the second thioether bond in the latter type of protein has been shown to cause little perturbation to the overall structure of the cytochrome [31]. However, although HCCS can in principle form the ‘trypanosome’ type cytochrome *c*, with one thioether bond those organisms lack the HCCS system but contain a putative novel System V for cytochrome *c* maturation. Interestingly, a recent study has shown that the cytochrome *c* maturation system in those organisms cannot produce a correct version of a CXXCH-containing cytochrome [32], thus adding to the evidence that a system distinct from HCCS is functional in that group of organisms and that HCCS needs the entire CXXCH motif for optimal performance*.* The failure of heme to attach to the H19M, H19K and H19R variants further demonstrates the importance of the histidine residue and thus it seems that the HCCS system cannot process CXXCR. No cytochrome *c* biogenesis specific system, except specific variants of Systems I or II will recognise anything except histidine.

### 4.2 C-terminal region of S. cerevisiae cytochrome *c* not required for maturation

Detectable amount of two truncated forms of *S. cerevisiae* cytochrome *c* were observed. The absence of reproducible heme attachment of the shortest fragment truncated, G28X, is probably due to degradation. The holocytochrome levels for the two longer fragments are significantly lower than the wild type, probably due to the reduced stability, and thus degradation of these N-terminal fragments of cytochrome *c*. The heme attachment to the truncated protein is consistent with the recent demonstration that heme was attached to an N-terminal fragment fused to a PDZ domain [33]. The truncation experiments also add to the evidence that the methionine residue M83, an axial ligand to the heme iron in holo cytochrome *c*, is not required for HCCS activity; it has been reported earlier that the M83A protein is readily produced by HCCS [34], certainly to a greater extent than the truncations.

### 4.3 The conserved phenylalanine and its spacing from CXXCH in the N-terminal region of *S. cerevisiae* cytochrome *c*

Within the N-terminus of mitochondrial cytochrome *c*, the importance of the phenylalanine residue F15 (yeast numbering) for maturation has been firmly established. The formation of stable and folded F15A (or F11A for the horse heart and human proteins) cytochrome *c* through periplasmic maturation using the Ccm system has shown that, contrary to a long-held assumption, the conserved F at position 11 is not crucial for the fold of the protein but rather for the action of HCCS. The consequences of substitution of F15 led to mixed results – F15Y variant retained a similar (80–90%) level, as judged by pyridine hemochrome analysis of holocytochrome production as the wild-type, whereas F15W and F15I variants had levels around 40% and 20% of that in the wild-type. One possible explanation for these observations is the aromatic nature of the R-groups of tyrosine (substantially) and tryptophan (moderately so) are recognised by HCCS and are compatible with a stable folded holo-cytochrome *c*. The bulky isoleucine is less successful in imitating phenylalanine in tertiary structure. Nevertheless, F is the preferred residue, on the basis of experiments described in the present paper, and that can explain its conservation. Glutamate and alanine, with very different R-group characteristics, are not able to replace the aromatic phenylalanine, hence holocytochrome production was abolished. However, as the presence of high levels of apocytochromes F15E and F15A variants demonstrates, these apo forms were available as potential substrates for HCCS and thus the absence of cytochrome maturation in these variants may be explained by lack of substrate recognition by HCCS. Our finding that the Y15F variant is well tolerated by HCCS from yeast is of interest in respect of the recent report of a similar finding for human HCCS [30]. In the latter case it was speculated that this might be an adaptation in the human enzyme to allow it to recognise both cytochrome *c* and cytochrome *c_1_*, the latter having Y at the corresponding position. However, our work shows that even though yeast has a dedicated enzyme for cytochrome *c*_1_ assembly the HCCS can still tolerate Y in that position.

The importance of an F residue at position 15 (yeast numbering) is now well established but it has been noted that apocytochromes *c*_2_ of both *P. denitrificans* and *Rhodobacter capsulatus* each contain an F residue, but at the equivalent of position 16; these apo proteins are not matured by HCCS [30], [35]. We found that deleting a K or a T between the F and the CXXCH motif in the mitochondrial cytochrome, and thus making the spacing similar to the bacterial proteins, attenuated the production of holo cytochrome *c* by HCCS but far from abolished it. Other had we found that inserting an alanine in the *P. denitrificans* sequence between the F and the CXXCH did not confer recognition by HCCS (unpublished results), which agrees with the results of a similar experiment [30] using human HCCS and *R. capsulatus* cytochrome *c*_2_. However, in two recent related studies that appeared after completion of the present work, recognition of a bacterial (*R. capsulatus*) cytochrome *c* by HCCS could be achieved either by a K11 insertion between in combination with an E9L substitution [35] or insertion of an A together with mutations of two glutamate residues at the N-terminus (E8K_E10I_Ala) in *R. capsulatus* cytochrome *c*_2_ [30]. In both the latter cases the spacing between F and CXXCH was increased to three residues but, there are clearly additional factors at work than just the spacing difference in different cytochrome amino acid sequences. Thus, it could be suggested that the phenylalanine residue and spacing in the N-terminal region of cytochrome *c* are both essential for optimal heme attachment performance by HCCS, although some heme attachment can occur with a spacing of two. The shift in wavelength of the alpha peak of the spectra of the deletion variants of *S. cerevisiae* indicates a change of nature in heme binding to the CXXCH motif locally, due to the change in spacing between residues F15 and C19.
